# Distributed Learning for Dynamic Channel Access in Underwater Sensor Networks

**DOI:** 10.3390/e22090992

**Published:** 2020-09-07

**Authors:** Huicheol Shin, Yongjae Kim, Seungjae Baek, Yujae Song

**Affiliations:** 1 Ocean Science and Technology (OST) School, Korea Maritime and Ocean University, Busan 49112, Korea; shc0305@kiost.ac.kr; 2 Maritime ICT R&D Center, Korea Institute of Ocean Science and Technology (KIOST), Busan 49111, Korea; yongjaekim@kiost.ac.kr (Y.K.); baeksj@kiost.ac.kr (S.B.)

**Keywords:** acoustic communication, deep reinforcement learning (DRL), distributed algorithm, dynamic channel access, multi-agent RL, underwater sensor networks

## Abstract

In this study, the problem of dynamic channel access in distributed underwater acoustic sensor networks (UASNs) is considered. First, we formulate the dynamic channel access problem in UASNs as a multi-agent Markov decision process, wherein each underwater sensor is considered an agent whose objective is to maximize the total network throughput without coordinating with or exchanging messages among different underwater sensors. We then propose a distributed deep Q-learning-based algorithm that enables each underwater sensor to learn not only the behaviors (i.e., actions) of other sensors, but also the physical features (e.g., channel error probability) of its available acoustic channels, in order to maximize the network throughput. We conduct extensive numerical evaluations and verify that the performance of the proposed algorithm is similar to or even better than the performance of baseline algorithms, even when implemented in a distributed manner.

## 1. Introduction

With the emergence of the Internet of Things (IoT) integrating a large number of heterogeneous end systems, the marine industry has been reconstructing itself. Accordingly, the digitalization and modernization of marine applications via the rapid advancement of IoT technologies have been covered in recent literature [1,2,3,4,5].

For the realization of maritime IoT, acoustic communication has received considerable attention as a promising technology for the construction of underwater sensor networks, because it can cover a distance of several hundred meters unlike radio frequency, optical, or magnetic induction-based communication techniques. Therefore, underwater acoustic sensor networks (UASNs) are expected to support a variety of marine applications such as oceanographic data acquisition, environmental monitoring, climate information recording, disaster prediction, assisted navigation, military surveillance, and reconnaissance [6,7]. However, there are many unsolved challenges associated with UASNs. Compared to terrestrial wireless networks, underwater acoustic communication environment has unique characteristics such as propagation delay, severely impaired channel condition, limited available bandwidth, energy constraint [7,8,9,10,11,12,13].

Many researchers have been engaged in investigating UASNs for managing harsh underwater environments [14,15,16,17,18,19,20,21,22]. In [14,15,16], propagation models for underwater acoustic communication were investigated and characterized in terms of the attenuation, time-varying multipath, and Doppler effect. To demonstrate the time-varying characteristics of underwater environments, the authors of [17] performed link performance measurement experiments and demonstrated the relationship between the current velocity of water and link performance. In [18], the authors constructed a wide-area network containing four fixed sensor nodes, two autonomous underwater vehicles, and one mobile node mounted on a supporting research vessel, to measure the various metrics such as the channel impulse response, signal-to-interference-plus-noise ratio, round trip time, and probability of packet loss. In [19], a novel acoustic interference channel model was developed based on the fact that acoustic signals have inconsistent transmission ranges in the vertical and horizontal directions. The authors of [20] presented several techniques such as multichannel equalization, phase tracking, symbol synchronization, Doppler tracking, and spatial multiplexing in multiple-input multiple-output systems, from the point of view of signal processing. In [21], a novel time synchronization technique was presented for UASNs where the propagation delay could affect the synchronization accuracy. The authors showed that the proposed technique achieved more precise time-synchronization performance with minimal energy consumption, when compared to conventional techniques. In [22], slotted floor acquisition multiple access was proposed by adding multiple timeslots to limit the effect of propagation delay in underwater communication.

In underwater environment, as most of the underwater IoT devices are powered by batteries, energy-efficient operations should be considered in UASNs. Therefore, many studies have been investigated energy-efficient techniques for UASNs [23,24,25,26,27,28,29,30,31,32,33,34,35,36,37]. In [23,24,25], several medium access control (MAC) protocols were introduced to save energy by avoiding collisions and reducing the number of unproductive transmissions in UASNs. The authors of [26] studied the minimization problem of transmit power while guaranteeing connectivity between each node and data sink for the energy-efficient operation of UASNs. In [27,28], the density of data sinks was minimized while satisfying the desired quality of service when two different MAC protocols were considered for channel access. To implement the energy-efficient operation of UASNs with three-dimensional topology, the amount of redundancy in a fountain-code–based transmission as well as the density of data sinks were optimized in [29,30]. In [31,32,33,34,35,36,37], routing techniques and relay transmission protocols were proposed to improve the energy efficiency for multi-hop relay UASNs. Especially, the authors of [31] analyzed the energy consumptions of acoustic communication modems in various states (i.e., transmit, receive, and idle), and an energy-efficient routing algorithm was proposed by optimizing the hop length based on this analysis. In addition, in [32], a pressure-gauge-information-based routing protocol, which exploited periodic beaconing to build directional trails toward the surface and featured greedy opportunistic directional forwarding for packet delivery, was developed. More details on underwater routing protocols can be found in the survey papers [38,39].

In the conventional wireless sensor networks (WSNs), one of the important issues is to the manner of accessing or assigning channels [40,41,42]. In [43], a multichannel protocol was proposed for WSNs, assuming that each IoT device was equipped with a single transceiver and that the MAC layer packet size was very small. In [44], a novel tree-based multichannel protocol based on a distance-aware interference model was presented and demonstrated through both simulations and real experiments for WSNs. The authors of [45] developed a channel allocation algorithm that could reduce the overhead of multichannel interference measurement by exploiting the power spectral density of the transmitter in low-power WSNs. However, all algorithms in [43,44,45] were centralized, and therefore were not suitable for UASNs because of the difficulty of maintaining a central entity that could coordinate IoT devices in underwater environments.

Many channel allocation techniques for UASNs have been studied extensively, and a number of optimal and suboptimal solutions have been presented [46,47,48,49,50,51,52]. In [46], a channel sharing technique that takes advantage of long delays of underwater channel was presented, and it resulted in an improved spectrum efficiency compared to the conventional spectrum reuse scheme. The authors of [47] proposed a heuristic channel allocation method that provides performance improvements in terms of minimum capacity and fairness. In [48], a channel selection scheme for cognitive UASNs was investigated to increase fairness and maximize the minimum capacity based on user location information. In addition, the authors of [49] studied a joint channel and power allocation algorithm for cognitive UASNs that aim at providing efficient spectrum utilization while avoiding harmful interference to other UASNs. In [50], the problem of minimization of total collision-free transmission time was demonstrated as NP-hard, and therefore the authors proposed a suboptimal algorithm which could work much better than the conventional scheduling algorithms. In [51], a joint channel and power allocation algorithm was developed to maximize the network capacity. This algorithm could be operated in a distributed manner and had no overhead when compared to the conventional MAC protocols. In [52], a receiver-initiated spectrum management system was presented wherein receivers replaced the role of transmitters in conventional protocols as the initializers of the handshake process. By collecting the local sensing results from the neighboring transmitters, the receivers could assign vacant spectrum resources and optimal transmit powers. Recently, with the development of deep reinforcement learning (DRL) algorithms, dynamic DRL-based resource management problems were investigated in UASNs [53,54]. In [53], an agent node which uses DRL-based MAC protocol learns underwater environment and occupy the spare time slots to achieve minimum collision when coexisting with a time division multiple access based node and a slotted ALOHA-based node. The authors of [54] proposed a DRL-based multiple access protocol which maximizes the occupation of available time slots caused by long propagation delay or not used by other nodes. Despite the extensive amount of existing studies, including the works in [46,47,48,49,50,51,52,53,54] on dynamic channel access for UASNs, there is no existing work that considers autonomous channel access with no coordination between underwater sensors and utilizing the prediction information of link qualities of available acoustic channels at the same time.

Hereafter, we focus on the detailed data communication scenario from each underwater sensor to its associated data sink (e.g., receiver) in UASNs to explicitly clarify the problems addressed in this work. In UASNs, when transmitting data from underwater sensors to a data sink, two approaches might be considered to manage the channel access between the sensors: The first approach is one wherein a data sink allocates the available acoustic channels to its associated underwater sensors in a centralized manner. However, generally, the data sink does not feature the functionality of radio resource management, unlike a base station in cellular networks [29]. The second approach is one wherein underwater sensors associated with the same data sink can cooperate with each other to share the channels; however, it is impractical to coordinate or exchange messages between the sensors to manage channel access in general UASNs [55]. In addition, it is difficult for each sensor to predict the link qualities of available acoustic channels because of the influence of a variety of factors such as pressure, density, salinity, and temperature, compared to terrestrial radio frequency channels. To overcome the limitations of UASNs described above, our idea is that each underwater sensor enables the simultaneously learning of both the behaviors (i.e., channel choices) of other sensors and the time-varying dynamics of available acoustic channels in a distributed manner, through learning the relationship between its action choice and the corresponding reward (i.e., RL). For this, we formulate a dynamic channel access problem of UASNs as a multi-agent Markov Decision Process (MDP). In particular, for reflecting the above idea, we design a state space of each sensor, which includes its previous action (i.e., previous channel choice), the estimation of channel error probabilities for all available channels, and two-bit feedback information corresponding to the previous action from its associated data sink. Note that adopting the two-bit local feedback mechanism enables each sensor to estimate the channel error probabilities of available acoustic channels itself. Then, we propose a distributed DRL-based algorithm, under which each sensor can choose the proper channel while avoiding not only the same channel selection with other sensors, but also the channels with the bad channel qualities without any coordination of other sensors.

We summarize the contributions of this work below.

- We formulate the dynamic channel access problem in UASNs as a multi-agent MDP, wherein each underwater sensor is considered an agent whose objective is to maximize the total network throughput without coordinating with or exchanging messages among underwater sensors.
- We propose a dynamic channel access algorithm for UASNs, based on deep Q-learning. In the proposed algorithm, each agent (i.e., underwater sensor) exploits partial information, i.e., only the feedback information between a data sink and that particular underwater sensor instead of complete information on the actions of all other agents, to learn not only the behaviors (i.e., actions) of the other sensors but also the physical features, i.e., channel error probability (CEP) of its available acoustic channels. This property ensures that each underwater sensor can implement the proposed algorithm in a distributed manner, i.e., there is no need for cooperation between agents.
- Through performance evaluations, we demonstrate that the performance difference between the proposed algorithm and the centralized algorithms is not that large, even though if it is implemented in a distributed manner. Moreover, it is identified that the performance of the proposed algorithm is much better than that of the random algorithm.

## 2. System Model

**Figure 1:**
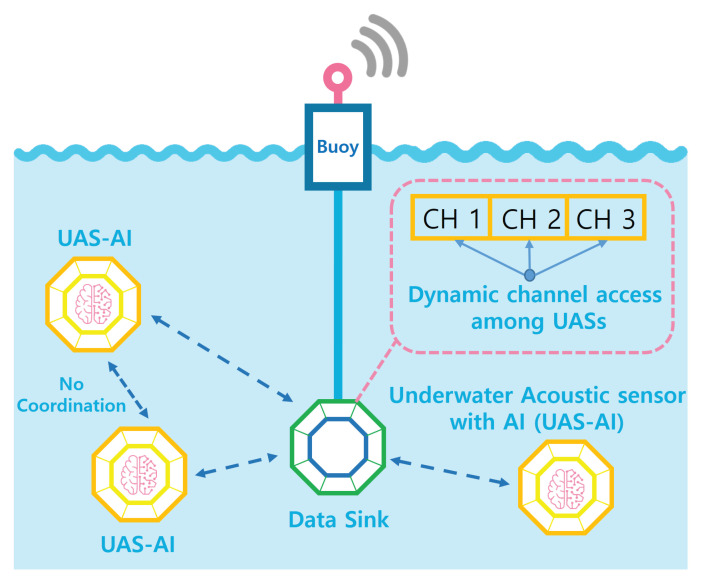
Illustration of underwater acoustic sensor networks (UASNs).

We consider UASNs that consist of a data sink and a set of underwater sensors $U=\left\{1,2,...,\left|U\right|\right\}$, as illustrated in Figure 1, where $\left|\cdot \right|$ denotes the cardinality of a set.

In the UASN, each underwater sensor collects a variety of information with respect to the underwater conditions, such as oceanographic data, ocean sampling data, environmental monitoring data, etc. It then sends the collected data to a data sink via an underwater acoustic link. The data sink gathers the data transmitted from the sensors and sends the aggregated data to a surface buoy through high-speed wired communication (e.g., wired fiber optical communication). Finally, the surface buoy, which is equipped with a radio frequency transceiver, transfers the data to a control center located on land. In this entire process of data transmission from the underwater sensors to a control sensor, this work focuses on the data transmission from the underwater sensors to the data sink via acoustic links.

The time domain of the underwater acoustic link is divided into time slots, and each time slot is utilized for the transmission of one packet. At the beginning of every time slot, each underwater sensor must choose one channel from among the shared acoustic channels, denoted by $K=\left\{1,2,\ldots ,\left|K\right|\right\}$, for packet transmission. It is assumed that the underwater sensors always have packets to transmit.

Among a variety of MAC protocols, we consider a random access protocol, which is the representative MAC protocol adopted in UASNs. The transmission of underwater sensor *u* on channel *k* can be successful if and only if sensor *u* alone occupies channel *k* (i.e., there is no collision) and there is no channel error on channel *k* due to bad channel quality in a given time slot. After the data transmission, sensor *u* receives feedback information from the data sink, which indicates whether the transmitted data have been delivered successfully to the data sink or not. In this work, we consider a two-bit feedback scenario. Let $o_{u}\left(t\right)$ be the feedback information of sensor *u* at time slot *t*, which is defined as follows,

(1) $$\begin{array}{c} o_{u}\left(t\right)=\left\{\begin{array}{cc} 0, & \mathrm{if}\;\mathrm{transmission}\;\mathrm{has}\;\mathrm{succeeded},\\ 1, & \mathrm{if}\;\mathrm{channel}\;\mathrm{error}\;\mathrm{has}\;\mathrm{occurred},\\ 2, & \mathrm{if}\;\mathrm{collision}\;\mathrm{has}\;\mathrm{occurred}. \end{array}\right. \end{array}$$

More specifically, if a packet has been delivered successfully, then $o_{u}\left(t\right)=0$. On the other hand, if the transmission has failed owing to channel error due to bad channel quality without collision, then, $o_{u}\left(t\right)=1$. Otherwise, $o_{u}\left(t\right)=2$, i.e., the transmission has failed due to collision. We differentiate three types of feedback because, when packet collision occurs (i.e., $o_{u}\left(t\right)=2$), it will be impossible to know at the data sink whether a channel error has occurred or not, regardless of the occurrence of an actual channel error. In this case, by feeding back this fact to the underwater sensor, the estimation of CEP is not updated, which will be explained in a next section.

## 3. Problem Formulation with MDP

In this section, we formulate a dynamic channel access problem for each underwater sensor, as an MDP. To determine the channel that each underwater sensor accesses in each time slot, we define an MDP with a tuple (S, A, *r*), where S is the state space, A is the action space, and *r* is the reward. The details of these parameters are explained subsequently.

**Figure 2:**
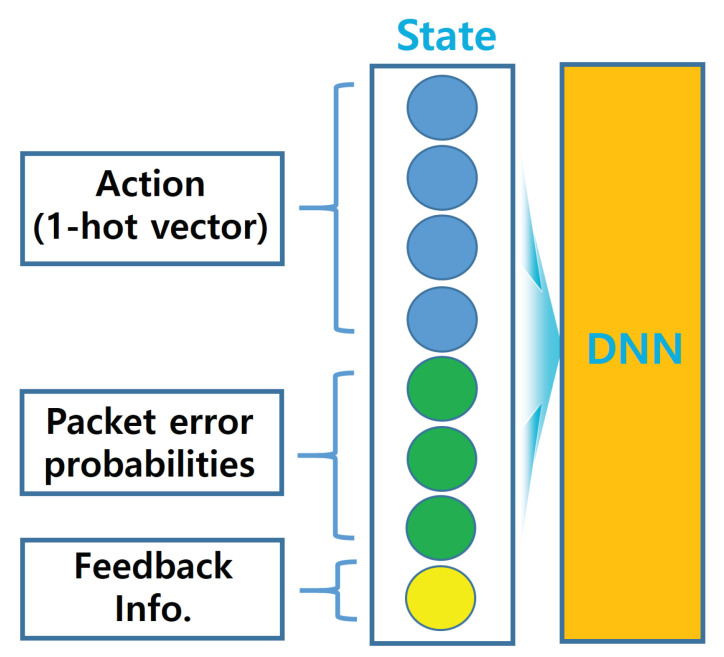
Illustration of state.

State $s_{u}\in S$ of sensor *u* can be expressed as a vector of size 2K+2, which is illustrated in Figure 2.

The first K+1 elements, which are presented as a one-hot vector of state $s_{u}$, stand for the action of sensor *u* executed at time t−1. Specifically, if sensor *u* does not transmit to a data sink in the time slot t−1, the first element is set to 1 and the remaining elements are set to 0. If the sensor transmits on channel *k* at time slot t−1, the (k+1)-th element is set to 1, and the remaining elements are set to 0. The following K elements of state $s_{u}$ are the estimation of the CEP of each channel k∈K for sensor *u*, conditioned on the event that the channel is idle. Note that the estimation of CEP on channel *k* for sensor *u*, denoted by ${\bar{p}}_{u}^{k}\left(t\right)$, is updated only after transmission on channel *k*, by using the feedback information as follows,

(2) $$\begin{array}{c} {\bar{p}}_{u}^{k}\left(t\right)=\left\{\begin{array}{cc} \alpha {\bar{p}}_{u}^{k}\left(t-2\right)+\left(1-\alpha \right)0, & \mathrm{if}\;\;a_{u}\left(t-1\right)=k,\;\;o_{u}\left(t-1\right)=0,\\ \alpha {\bar{p}}_{u}^{k}\left(t-2\right)+\left(1-\alpha \right)1, & \mathrm{if}\;\;a_{u}\left(t-1\right)=k,\;\;o_{u}\left(t-1\right)=1, \end{array}\right. \end{array}$$

where $\alpha \in \left[0,1\right]$ is the moving rate and $a_{u}$ is the action of sensor *u*, which will be explained below. Note that when collision occurs (i.e., $o_{u}\left(t\right)=2$) on channel *k*, the estimation of CEP is not updated. As mentioned before, this is because, when a collision occurs, the data sink will not be able to judge whether a channel error has occurred or not. The last element of state $s_{u}$ is the feedback information received after transmission at time slot t−1, described in (1).

An action $a_{u}\in A$ is an element of the action space A, which is the set of available actions that the underwater sensor *u* can choose, as given by

(3) $$A\left(t\right)=\left\{0,1,2,\ldots ,K\right\},$$

where $a_{u}\left(t\right)=0$ means that a sensor does not transmit a packet at time slot *t*, and $a_{u}\left(t\right)=k$ means that the sensor transmits a packet on channel *k* at time slot *t*. Note that the reward of each underwater sensor depends on not only its action, but also other sensors’ actions, which constitute the unknown network environment that each sensor must learn. As such, the action profile of sensor *u* for all sensors except itself at time *t* can be defined as follows,

(4) $$a_{-u}\left(t\right)={\left\{a_{i}\left(t\right)\right\}}_{i\neq u}.$$

Finally, we can define a reward function $r\left(t\right)$ as follows,

(5) $$\begin{array}{c} r_{u}\left(t\right)=\left\{\begin{array}{cc} 1, & \mathrm{if}\;\;o\left(t\right)=0,\\ 0, & \mathrm{Otherwise}, \end{array}\right. \end{array}$$

where underwater sensors that receive $o\left(t\right)=0$ from the data sink can achieve a positive reward, i.e., r(t)=1. With the help of (5), the total network throughput, which is the performance metric in this work, can be presented as follows,

(6) $$r_{net}\left(t\right)=\sum_{u\in U}r_{u}\left(t\right).$$

As this work focuses on sequential decision making for dynamic channel access in UASNs, both the immediate and future rewards should be considered when making a decision. As such, we define the accumulated discounted total network throughput as follows,

(7) $$R\left(t\right)=\sum_{t=1}^{T}\gamma^{t-1}r_{net}\left(t\right),$$

where γ∈[0,1] is a discount factor that determines the effect of the future reward.

To maximize the total network throughput in UASNs, two approaches can be considered to manage the channel access between the sensors. The first approach is to allocate the available acoustic channels to its associated underwater sensors from data sinks in a centralized manner. However, data sinks do not have the ability to manage radio resources and only receive data from sensors. The second approach is to cooperate between data sinks by sharing channel information. The cooperation or exchanging messages, however, is unrealistic in general UASN environment. To reflect such characteristics, this work considers a distributed UASN scenario, where each sensor determines its transmission channel by partially observing the actions of other sensors (i.e., $a_{-u}\left(t\right)$) with the help of local feedback information, i.e., $o_{u}\left(t\right)$, from the data sink.

## 4. Background on Q-Learning and Deep Reinforcement Learning

In this section, we present a brief background on Q-learning and DRL, which will be utilized to develop the proposed algorithm in next section. To avoid clutter of indices, we assume a fixed typical sensor (say sensor *u*) and drop the sensor indices for all the parameters.

Q-learning is a reinforcement learning algorithm that can help find the best policies (i.e., a sequence of actions over time) for dynamic programming problems. Because the expected reward can be calculated from among the available actions without prior knowledge on the environment, Q-learning has been widely adopted in a variety of decision-making applications. More specifically, we denote $Q_{\pi }\left(s,a\right)$ as the Q-function given policy π where a state–action pair $\left(s,a\right)$ is considered as a variable. The Q-function is defined as the sum of discounted rewards achieved when action *a* is taken in the initial state s under policy π, and it can be presented in a recursive form:

(8) $$Q_{\pi }\left(s,a\right)=r\left(s,a\right)+\delta \sum_{s^{\prime }\in S}\sum_{a^{\prime }\in A}P_{{\mathrm{ss}}^{\prime }}^{a}Q_{\pi }\left(s^{\prime },a^{\prime }\right),$$

where $\delta \in \left[0,1\right]$ is the discount factor determining the effect of the future rewards, $P_{ss^{\prime }}^{a}$ is the state transition probability from state s to state $s^{\prime }$ by action *a*, and $\left(s^{\prime },a^{\prime }\right)$ is the next state–action pair when an agent executes action *a* in state s.

The agent aims at finding the optimal policy $\pi^{*}\left(s\right)$ that maximizes (8) for each state s. We denote $Q_{\pi^{*}}\left(s,a\right)$ as the Q-function for the state–action pair $\left(s,a\right)$ under the optimal policy $\pi^{*}\left(s\right)$, such that (8) can be rewritten as

(9) $$Q_{\pi^{*}}\left(s,a\right)=r\left(s,a\right)+\delta \sum_{s^{\prime }\in S}\sum_{a^{\prime }\in S}P_{ss^{\prime }}^{a}Q_{\pi^{*}}\left(s^{\prime },a^{\prime }\right),$$

where an optimal policy can be obtained as $\pi^{*}\left(s\right)=\arg\max_{a\in A}\left[Q_{\pi^{*}}\left(s,a\right)\right],\;\;\;\;forall\;s\in S$.

However, for some systems, we cannot calculate $Q_{\pi^{*}}\left(s,a\right)$ from (9), because the state transition probability $P_{ss^{\prime }}^{a}$ is practically unknown to the agents. Q-learning has been widely adopted as an alternative [56], as it is one of the representative model-free algorithms. Q-learning constructs a $\left|S\right|\times \left|A\right|$ Q-table, each element of which is a Q-value. The agents can update each element $Q\left(s,a\right)$ in the Q-table using the following equation,

(10) $$Q\left(s,a\right)=\left(1-\alpha \right)Q\left(s,a\right)+\alpha \left[r\left(s,a\right)+\delta \max_{a^{\prime }\in A}Q\left(s^{\prime },a^{\prime }\right)\right],$$

where α is the learning rate.

Note that Q-learning works well if the state–action space is small, whereas it becomes impractical if the size of the state–action space increases, because of two reasons: First, many state–action pairs in the state–action space are visited very rarely, which degrades the performance considerably. Second, we need a Q-table that can hold all Q-values corresponding to the state–action pairs in the space, which might make the storage complexity intolerable.

To overcome these issues, a potential was proven by DRL that combines deep neural network (DNN) with Q-learning, which is also called Deep Q-Network (DQN) [57]. Using DQN, the DNN maps a partially observed state to an action, by replacing the infinitely large Q-table with a relatively small DQN and storing the weights of the DQN in a local memory. Because of this feature, large-scale models can be represented using the DNN, and the algorithm will have the ability to maintain good performance for large-scale models. Further, a variety of DQN variants have been suggested in recent years [58,59,60]. The first and simplest form for a variant of DQN is double DQN, which is called DDQN, introduced in [58]. The key idea of DDQN is to separate the selection of greedy action with action evaluation. As such, DDQN expects to reduce the overestimation of Q-values in the training process. The work in [59] presents a prioritized experience replay that gives priority to a sample based on its absolute value of time-difference error. It is proven that prioritized experience replay combining with DDQN offers stable convergence of policy network and achieves a performance up to five times greater than DQN with respect to normalized mean score on 57 Atari games. In [58], the network architecture called dueling network is investigated. Under dueling architecture, there are two collateral networks that coexist: one network parameterized by θ estimates state-value function, and the other parameterized by $\theta^{\prime }$ estimates advantage action function. The two networks are then aggregated to approximate Q-value function. The detailed description on such DQN variants are explained in Section 3.2 of the work in [61].

## 5. Proposed Algorithm

In this section, we propose a multi-agent DQN-based dynamic channel access algorithm for distributed UASNs, as illustrated in Algorithm 1. As mentioned before, it is impractical to coordinate or exchange messages between underwater sensors to manage channel access in UASNs. Thus, we aim at developing a distributed algorithm that does not require coordination among underwater sensors. Algorithm 1 presents the proposed algorithm, which is implemented in each underwater sensor.
**Algorithm 1** DQN-based dynamic channel access algorithm for each underwater sensor1: Establish a trained DQN with weights θ and a target DQN with weights $\theta^{-}$2: Initialize θ and set $\theta =\theta^{-}$3: In time slot t≤Z, the agent randomly selects an action *a* and executes the action, and then observes the reward *r* and new state $s^{\prime }$4: Store $\left(s,a,r,s^{\prime }\right)$ in reply buffer D5: Repeat:6: **for**
t≥Z to *T*
**do**7:    In each time slot *t*, the agent chooses action $a\left(t\right)$ by following the below distribution described in Equation (11)8:    Execute $a\left(t\right)$ and observe reward $r\left(t\right)$, feedback information $o\left(t\right)$ and new state $s^{\prime }\left(t+1\right)$9:    Store $\left(s\left(t\right),a\left(t\right),r\left(t\right),s^{\prime }\left(t+1\right)\right)$ in reply buffer D10:  Update the estimation of corresponding to the chosen action using (2) with feedback information $o\left(t\right)$11:  The agent randomly samples a minibatch with *Z* experiences from reply buffer D, and then updates weights θ for the trained DQN12:  In every predetermined time slot, the agent updates the weights for the target DQN with $\theta^{-}=\theta$13: **end for**


In Algorithm 1, at each time *t*, the agent chooses $a\left(t\right)$ according to the following distribution,

(11) $$\mathrm{Pr}\left(a\left(t\right)=a\right)=\frac{\left(1-\gamma \right)e^{\beta Q\left(a\right)}}{\sum_{\tilde{a}\in A\left(t\right)}e^{\beta Q\left(\tilde{a}\right)}}+\frac{\gamma }{K+1},$$

where β is the temperature and $\gamma \in \left[0,1\right]$ is the weighting factor for determining an action. It should be noted that (11) balances between the softmax and ε-greedy methods, which is known as the Exp3 scheme [62]. Specifically, if γ is set to 1, the action is selected randomly, by following a uniform distribution. As γ goes to 0, the algorithm becomes greedier with time, with respect to selecting actions with high estimated Q-values.

## 6. Performance Evaluations

### 6.1. Network Environment

We conducted performance evaluations to identify the validity of the proposed algorithm. For the performance evaluations, we adopted the Bellhop channel model for generating the underwater acoustic channels, which was introduced in [63]. This channel model reflects the large-scale effects due to path-loss and multiple propagation paths as well as the small-scale effects such as scattering in UASNs. The ambient noise of UASNs in kHz was modeled as $10\log N\left(f\right)=N_{1}-\tau \log\left(f\right)$, where $N_{1}$ and τ were set to 50 dB re micro Pa per Hz and 18 dB/decade, respectively, which were determined with empirical experiments [64]. Table 1 presents the system parameters adopted for performance evaluations.

### 6.2. Learning Environment

We adopted a DQN structure that was a fully connected neural network with two hidden layers containing 128 neurons. The hyperparameters for learning are presented in Table 2.

### 6.3. Baseline Schemes

For a comprehensive performance evaluation, we compare the performance, given in terms of total network throughput, of the proposed algorithm achieved from Algorithm 1 against those of three algorithms: the optimal algorithm, the random algorithm, the exact CEP algorithm. Similar to the work in [65], the optimal algorithm performs optimal channel allocation that maximizes total network throughput in a centralized manner. For this, it should be assumed that the data sink has the functionality of radio resource management and notifies the channel allocation result to its associated sensors. On the contrary, following the random algorithm [66], each underwater sensor chooses its transmission channel among available channels at each time slot in a fully distributed manner. That is, there is no need for a feedback process. The exact CEP algorithm is based on the proposed algorithm with a slight difference as follows. In the exact CEP algorithm, it is assumed that each underwater sensor can know the exact CEPs for all available channels at each time slot and utilizes those in the state space, and then chooses an action through Algorithm 1. For this, before transmitting sensing data, signal exchanges, such as the pilot transmission from each sensor to the data sink for all available channels, the link qualities estimation of the channels between the sensors and the data sink, and the feedback mechanism to report the results of the estimation, are needed. On the other hand, in the proposed algorithm, each sensor utilizes the estimated CEPs in the state space, which are computed by using only feedback information transmitted from the data sink, as described in (2).

**Figure 3:**
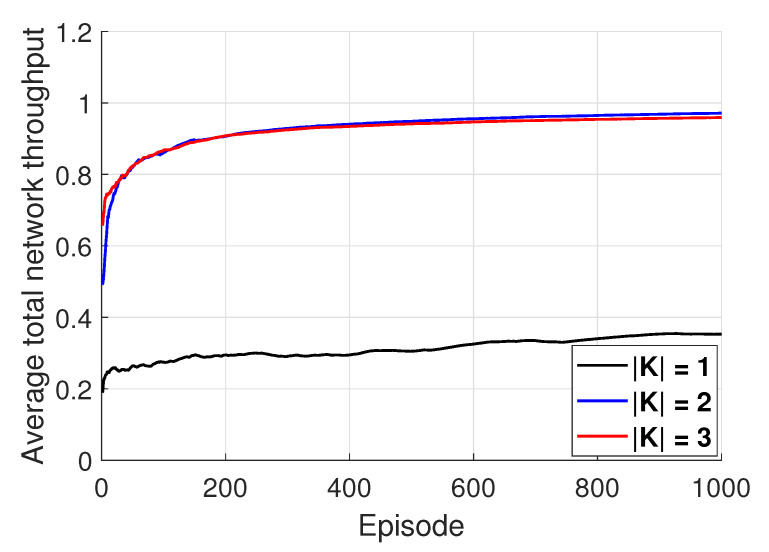
Illustration of the performance of the proposed algorithm.

Figure 3 shows the performance of the proposed algorithm under a change in the number of available channels $\left|K\right|$. In the figure, the x-axis represents an episode consisting of 500 timesteps and the y-axis represents the moving average of the total network throughput average achieved during an episode. From Figure 3, it can be identified that, regardless of $\left|K\right|$, when the proposed learning algorithm is executed at each sensor, the total network throughput improves and then converges at specific values over time. The performance of the proposed algorithm under $\left|K\right|=1$ is much lower than those under $\left|K\right|$ = 2 and 3. This is because, in the case of $\left|U\right|>\left|K\right|$, the probability of collision between underwater sensors increases and the total network throughput decreases, accordingly. In the case where sufficient channels are assigned to the sensors, i.e., if $\left|U\right|\leq \left|K\right|\;$, the performance of the proposed algorithm remains almost the same, regardless of $\left|K\right|$. This is because, although $\left|K\right|$ increases, the probabilities of collision will be same.

### 6.4. Performance Evaluations

**Figure 4:**
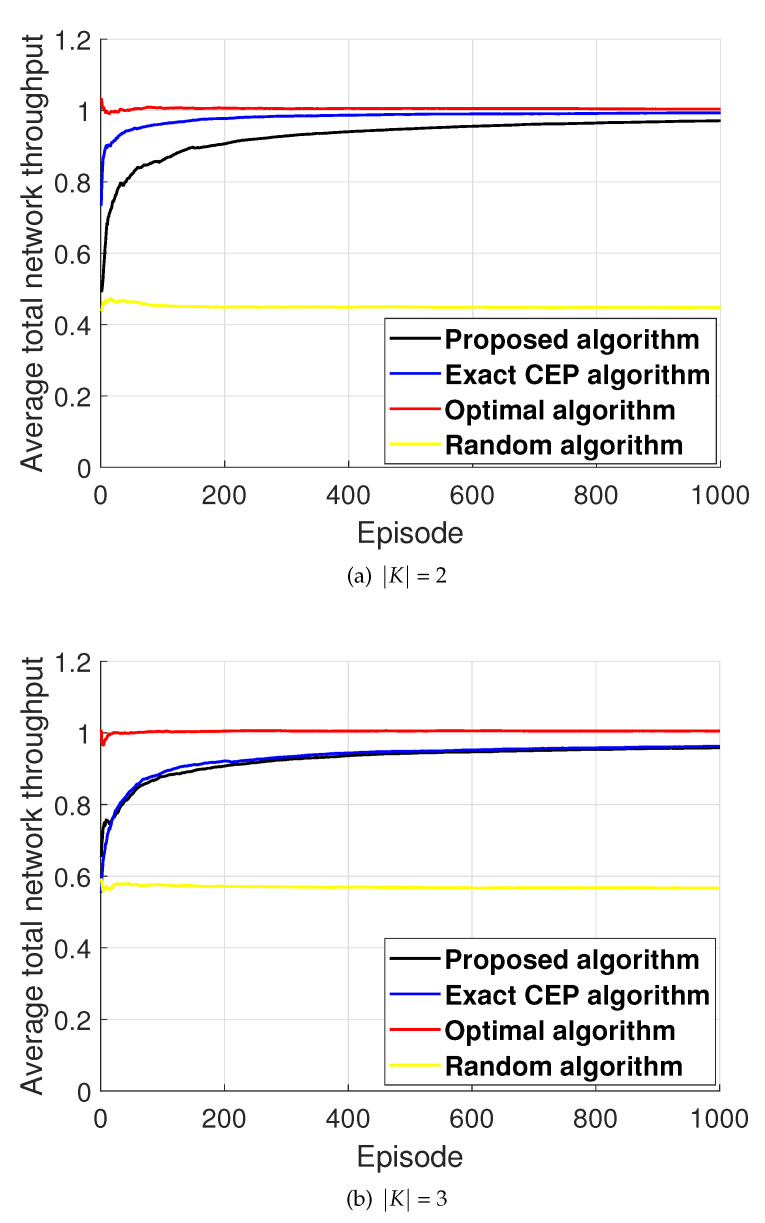
Illustration of performance comparison of the proposed and baseline algorithms.

Figure 4 presents the performance comparison of the proposed algorithm and the baseline schemes described above. Figure 4 illustrates that the proposed algorithm performs much better than the random algorithm. On the other hand, the performance of the proposed algorithm is lower than those of the exact CEP algorithm and optimal algorithm. Nevertheless, we emphasize that, to implement the exact CEP algorithm at each sensor, the exact information on the CEPs for all available channels should be notified, which might be impractical in the case of UASNs. In contrast, even though the proposed algorithm only exploits two-bit feedback information on the previous transmission result to estimate their CEPs, its performance is almost similar to that of the exact CEP algorithm. Moreover, the proposed algorithm shows at least 80 % performance, when compared to the performance of the optimal algorithm, which requires a fully centralized entity for assigning the channels to each sensor. These results illustrate the practical validity of the proposed algorithm.

**Figure 5:**
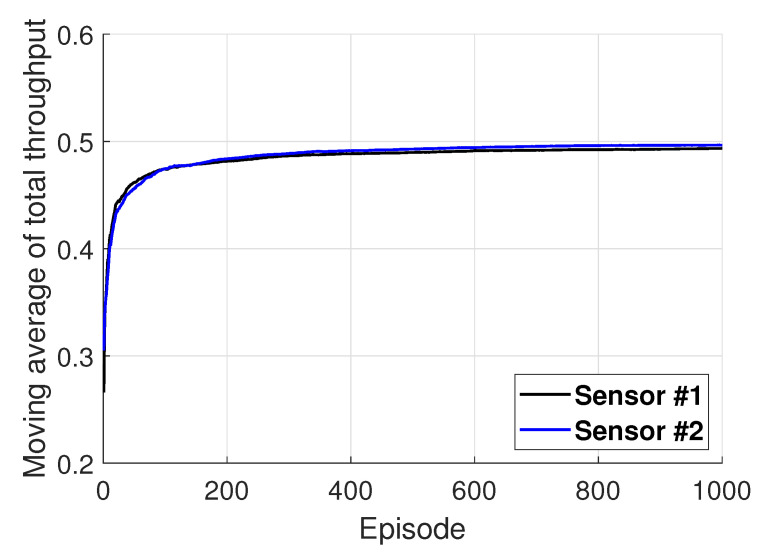
Illustration of the performance of each underwater sensor applying the proposed algorithm.

Figure 5 shows the performance of each underwater sensor under the application of the proposed algorithm under $\left|K\right|=2$. From Figure 5, it can be observed that, despite performing learning in a distributed manner, each sensor occupies the channel separately to avoid collisions, which results in the improvement of the total network throughput.

**Figure 6:**
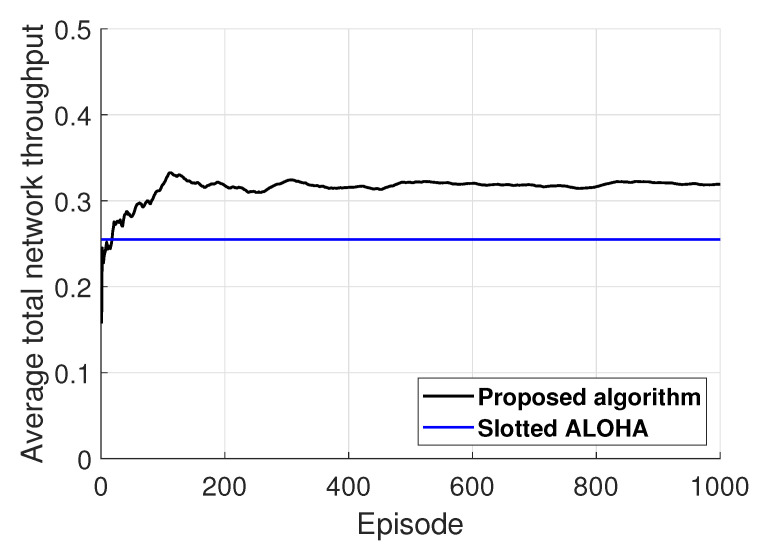
Illustration of performance comparison of the proposed and slotted ALOHA.

Figure 6 shows the performance comparison between the proposed and slotted ALOHA in considered UASNs with $\left|K\right|=1$. Under the slotted ALOHA, the number of backlogged queues in a data sink cannot exceed the number of associated sensors in a data sink, such that we set the channel access probability as $1/\left|U\right|$. As such, to implement the slotted ALOHA, the information on the number of underwater sensors associated with a same data sink is needed at each sensor. Figure 6 illustrates that the proposed algorithm outperforms better than the slotted ALOHA, not long after starting the learning of the proposed algorithm. This illustrates the validity of the proposed algorithm.

## 7. Conclusions

We proposed a multi-agent DQN-based dynamic channel access algorithm for distributed UASNs. The proposed algorithm helped each underwater sensor in the UASNs learn not only the behaviors (i.e., actions) of other sensors, but also the physical features (i.e., CEP) of its available acoustic channels. For this, we formulated the dynamic channel access problem of UASNs as an MDP, where each underwater sensor aimed to maximize the total network throughput without coordinating or exchanging messages among underwater sensors. Through performance evaluations, it was identified that the performance difference of the proposed algorithm, when compared to those of centralized channel access algorithms, was not very large, whereas the proposed algorithm performed much better than the random algorithm.

## Figures and Tables

**Table 1:** List of network parameters.

Network Parameter	Value
Number of active sensors and data sink	2, 1
Surface height (depth)	100 m
Height of sensors and data sink	10 m
Transmit power of sensors	20 W
Number of available acoustic channels	3
Minimum frequencies of available channels	[10, 30, 50] KHz
Bandwidth of each channel	10 KHz

**Table 2:** List of DQN hyperparameters.

Hyperparameter	Agent
Batch size	6
Optimizer	Adam
Activation function	Relu
Learning rate	$10^{-4}$
Experience replay size	1000
Discount factor η	0.99
